# Carotid Body Tumor: A Case Report and Review of the Literature

**DOI:** 10.7759/cureus.115359

**Published:** 2026-08-28

**Authors:** Rebeca Y Paredes, Edna M Arias Álvarez

**Affiliations:** 1 General Surgery, Institute for Social Security and Services for State Workers, Mazatlán, MEX; 2 Vascular Surgery, Institute for Social Security and Services for State Workers, Mazatlán, MEX

**Keywords:** angiotomografía, carotid body paraganglioma, carotid body tumor diagnosis, case report, glomus tumour

## Abstract

Carotid body tumors or carotid body paragangliomas, are rare, highly vascular neuroendocrine neoplasms originating from the paraganglionic cells of the carotid body situated near the adventitial layer of the carotid bifurcation. Although they typically exhibit slow growth and benign behavior, their close proximity to the neurovascular structures of the neck poses diagnostic and therapeutic challenges. Timely recognition through clinical examination and imaging studies is crucial for establishing the diagnosis and improving the prognosis. We present the case of an 84-year-old male with a progressively enlarging mass in the right parotid region, present for one month, without associated neurological symptoms. Carotid body tumors should be considered in the differential diagnosis of neck masses. Correlating clinical findings with imaging studies enables a timely diagnosis and appropriate treatment planning, thereby fostering a favorable clinical outcome.

## Introduction

Carotid body tumors are rare neoplasms with an incidence of one to two per 100,000 and account for 0.6% of the head and neck tumors that continue to pose a clinical diagnostic challenge due to their low incidence, slow growth, and typically asymptomatic presentation during the early stages. Carotid body tumors usually manifest as asymptomatic anterior neck masses. Large tumors can present with symptoms of a space-occupying lesion in this location, such as fullness, pain, dysphagia, odynophagia, hoarseness of voice, and stridor. Functional carotid body tumors may produce neuroendocrine secretions causing catecholamine-related symptoms such as palpitations, headaches, hypertension, tachycardia, or flushing. This insidious progression explains why diagnostic delay is frequently reported in the literature and underscores the importance of including this entity in the differential diagnosis of lateral neck masses, particularly in patients with lesions located at the carotid bifurcation and lacking signs of infection or obvious malignancy [1].

Clinical evaluation remains the first step in guiding the diagnosis. The presence of a firm cervical mass that is mobile laterally but has limited craniocaudal mobility, known as Fontaine's sign, is a finding suggestive of a carotid body tumor. However, since this characteristic is not always present, the definitive diagnosis relies on imaging studies. Doppler ultrasound is typically the initial study due to its availability and ability to demonstrate a solid, hypervascularized lesion; 4) Ultrasound, computed tomography (CT), and magnetic resonance imaging (MRI) are useful radiological tools in diagnosis. Yet angiography is essential to study the vascular anatomy. Furthermore, these modalities allow for the identification of the characteristic divergent displacement of the internal and external carotid arteries, known as the "lyre sign", as well as the recognition of patients with a possible hereditary origin through appropriate clinical and genetic correlation [2].

From a prognostic standpoint, carotid body tumors exhibit predominantly benign behavior and slow growth, with a low rate of malignant transformation. Prognosis depends primarily on tumor size, involvement of neurovascular structures, the presence of multifocal disease, and the patient's genetic background, rather than on histopathological characteristics, since there are currently no reliable pathological criteria to definitively distinguish between benign and malignant paragangliomas. A biopsy is contraindicated due to hypervascularity and the proximity of the tumor to various vascular and nervous structures [2, 3].

Recent studies also highlight the importance of multidisciplinary evaluation and comprehensive imaging characterization for establishing individualized management and follow-up strategies. Proper interpretation of preoperative studies allows for a more precise identification of the extent of anatomical involvement and an estimation of clinical prognosis, thereby facilitating more appropriate decision-making for each patient [4].

In the present case, the correlation between clinical findings and imaging studies enabled a timely diagnosis and adequate characterization of the lesion prior to determining the therapeutic approach. The absence of evidence suggesting multifocal disease or secretory activity, combined with the observed favorable clinical course, aligns with reports in the literature indicating that most carotid body tumors follow a satisfactory course when identified early and managed with appropriate clinical and imaging follow-up [1, 4].

## Case presentation

**Figure 1:**
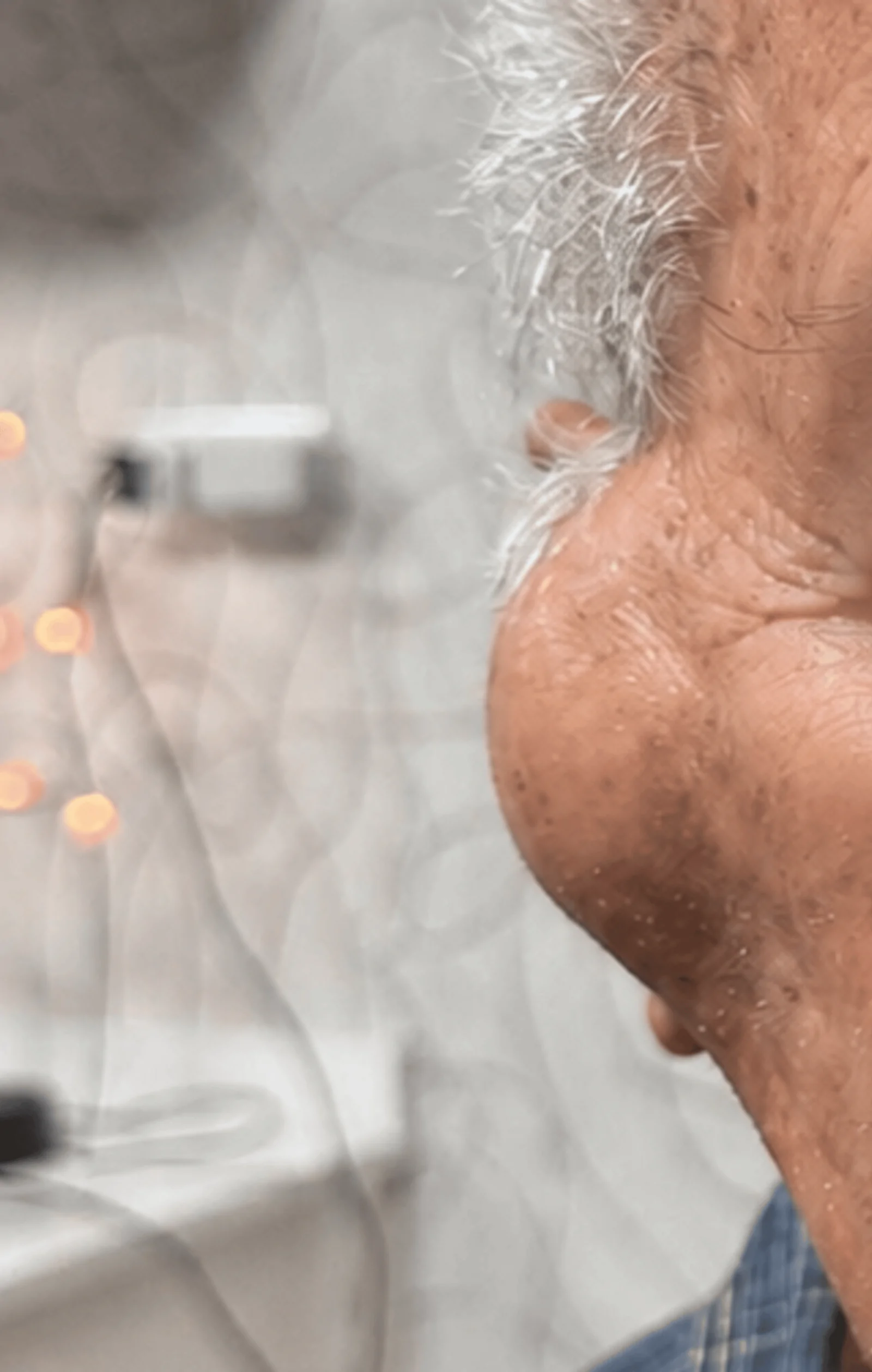
Representative image of the right paraganglioma lesion.

**Figure 2:**
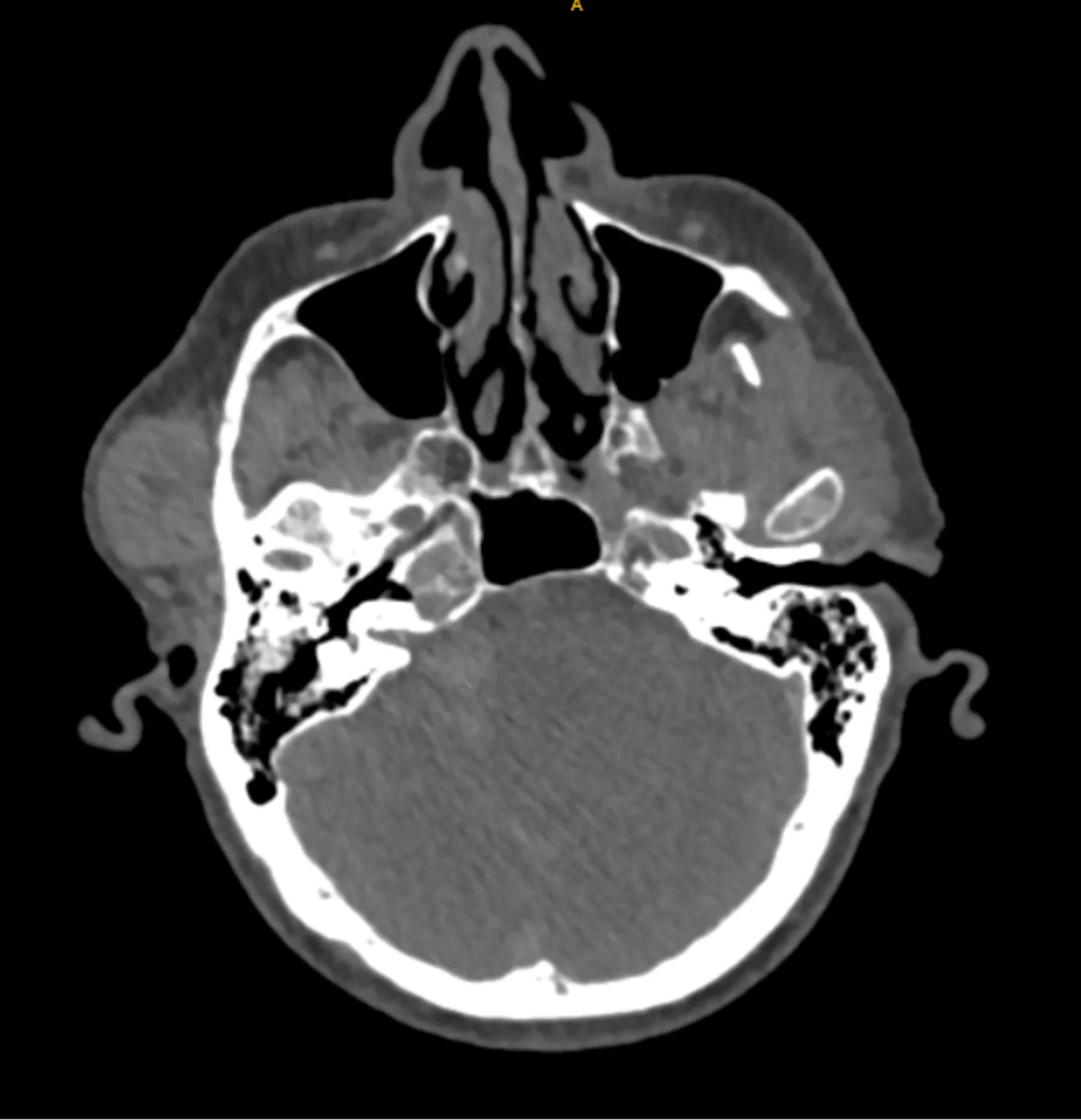
In the superficial lobe of the right parotid region, a rounded mass with lobulated borders is observed, measuring approximately 41 x 37 x 30 mm.

**Figure 3:**
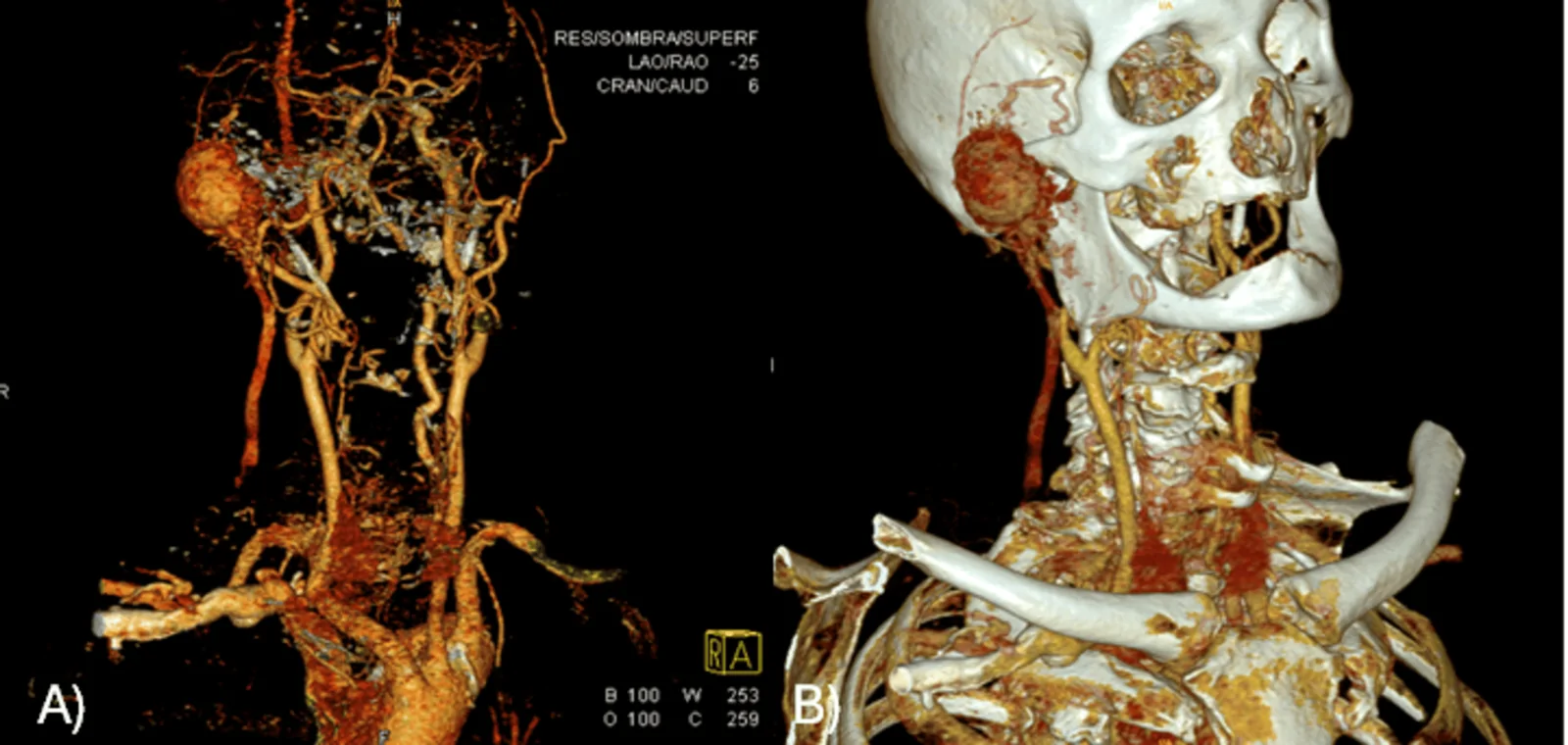
A rounded, hypervascular mass is observed with an internal hypodense area, surrounded by tortuous afferent vessels from the external carotid artery and tortuous efferent vessels draining into the right external jugular vein.

The patient was an 86-year-old male with a history of chronic arterial hypertension and a prior nephrectomy for a left kidney tumor. He denied substance abuse and had a known allergy to nonsteroidal anti-inflammatory drugs. He was referred to the Department of Angiology and Vascular Surgery due to a painful, "pulsatile," non-tender, palpable, semi-soft mass in the temporal region measuring approximately 7 cm x 3 cm, which had shown rapid enlargement over the course of one month (Figure 1). Differential diagnoses of carotid body tumors should be distinguished from masses in the neck or lesions originating from the carotid space. The most important differential diagnoses include aneurysm or pseudoaneurysm of the carotid artery, hematoma, glomus vagale tumor, and vagal schwannoma. A Doppler ultrasound revealed a temporal artery aneurysm; consequently, a thin-slice CT angiography with 3D reconstruction was ordered as part of the diagnostic workup (Figures 2, 3).

CT angiography revealed a rounded, hypervascular mass with lobulated margins, measuring approximately 41 x 37 x 30 mm and containing an internal hypodense area that exhibited intense early enhancement and rapid washout; the mass was surrounded by tortuous afferent vessels from the external carotid artery and tortuous efferent vessels draining into the right external jugular vein (Figure 3).

## Discussion

The carotid body is the largest collection of paraganglia in the head and neck and is found in the carotid space. Carotid body tumors were first described by von Haller in the year 1743. Carotid body tumors, also known as paragangliomas or chemodectomas, are rare neuroendocrine neoplasms that arise near the carotid bifurcation within glomus cells derived from the embryonic neural crest. The reported incidence of carotid body tumors is one to two per 100,000. Carotid body tumors are rare chemical receptor tumors, which account for 0.6% of the head and neck tumors in humans. Carotid body tumors are usually benign, with the incidence of malignant tumors below 10%; diagnosing them remains a clinical challenge due to their low incidence, slow growth, and typically asymptomatic course during the early stages. Most patients seek medical attention only when progressive neck swelling prompts a consultation, a pattern that contributes to diagnostic delay. Jiménez et al. describe this as the most common presentation and emphasize the importance of including this entity in the differential diagnosis of lateral neck masses, particularly those located at the carotid bifurcation [1].

More recent studies have provided greater insight into the biological behavior of these tumors. Head and neck paragangliomas have been associated with gene mutations in the succinate dehydrogenase complex, but the clinical significance remains unclear. These alterations are associated with bilateral disease, multifocality, and an increased risk of recurrence; consequently, genetic evaluation is currently recommended for young patients, those with a family history, and those with multiple paragangliomas [2].

Regarding diagnosis, Valderrama-Treviño et al. note that physical examination is the first step in clinical evaluation; however, diagnostic confirmation relies on imaging studies. Doppler ultrasound serves as a useful initial tool, while CT angiography and magnetic resonance imaging allow for the precise determination of tumor location, vascularity, and the relationship with the carotid arteries. Furthermore, they describe the "lyre sign" as one of the most characteristic radiological findings of carotid body tumors and highlight the utility of the Shamblin classification. In 1971, the Shamblin research group introduced a classification system according to the relationship with the carotid arteries in order to determine the resectability of these tumors. Shamblin type I tumors are small lesions at the carotid bifurcation and can generally be removed without difficulty. Shamblin type II tumors are larger and significantly splay the carotid bifurcation but do not circumferentially encase the carotid arteries. Shamblin type III tumors are large and encase the vessels and thus are the most difficult type to attempt resection on. According to the Shamblin classification, type III tumors are associated with more perioperative neurovascular complications and complex surgical procedures [3].

Similarly, Segovia-Vergara et al. emphasize that accurate imaging characterization facilitates clinical risk stratification and the planning of patient follow-up. Tumors classified as Shamblin I typically follow a favorable course, whereas type II and III lesions show greater involvement of the carotid bifurcation and a higher likelihood of neurological or vascular complications. Consequently, the Shamblin classification serves as a useful prognostic tool from the time of diagnosis [4].

In our patient, the correlation between the physical examination and CT angiography enabled a timely diagnosis and adequate characterization of the lesion prior to determining the therapeutic approach. The observed clinical course aligns with findings in the literature, where prognosis depends primarily on tumor size, neurovascular involvement, and the presence of multifocal disease, rather than on histopathological characteristics. Furthermore, clinical and imaging follow-up remains a fundamental component for detecting disease recurrence or progression, particularly in patients with hereditary risk factors [2, 4].

## Conclusions

Carotid body paraganglioma is a rare neoplasm that should be considered in the differential diagnosis of neck masses, particularly when rapid growth and hypervascular characteristics are present. Timely recognition of clinical manifestations, combined with imaging studies such as CT angiography, enables an accurate diagnosis, assessment of tumor extent, and prognostic evaluation. A carotid body tumor requires early diagnosis and an adequate multidisciplinary team. The diagnosis must be considered in the case of any pulsatile cervical mass. Surgery is the treatment of choice despite its risks, especially in large tumors. The therapeutic indication should, ideally, be set in a multidisciplinary consultation.
